# Scoliosis Following Chest Wall Resection for Tumor With and Without Prophylactic Fixation: Case Series

**DOI:** 10.7759/cureus.21115

**Published:** 2022-01-11

**Authors:** Varan Haghshenas, Michael Moghimi, Mimi P Haghshenas, Caleb Shin, Brendan M Holderread, Takashi Hirase, Darrell S Hanson, Laurence Rhines, Rex Marco

**Affiliations:** 1 Orthopedic Surgery, Houston Methodist Hospital, Houston, USA; 2 Orthopedics, Baylor College of Medicine, Houston, USA; 3 Radiology, Houston Methodist Hospital, Houston, USA; 4 Orthopedics and Sports Medicine, Houston Methodist Hospital, Houston, USA; 5 Neurosurgery, Monroe Dunaway (MD) Anderson Cancer Center, Houston, USA

**Keywords:** thoracic surgery, chest wall resection, chest wall resection complications, chest wall tumour, scoliosis

## Abstract

Posterior chest wall resection is a complex surgical procedure that involves removing any anatomical structure that surrounds the lungs and pleura, such as the intercostals, ribs, and soft tissues. The etiology of scoliosis that develops after chest wall excision is likely both mechanical and paralytic in nature. We report seven cases of scoliosis following posterior chest wall resection. Our results suggest that the prophylactic treatment of scoliosis after chest wall resection decreases the risk of scoliosis.

## Introduction

Chest wall resection is a complex surgical procedure that involves removing any anatomical structure that surrounds the lungs and pleura, such as the intercostals, ribs, and soft tissues. It is used for the treatment of several thoracic disorders, including, but not limited to, chest wall deformities and tumors. These tumors consist of primary or metastatic tumors of the soft tissue or bone and primary tumors of adjacent organs that invade the chest wall. Primary chest wall tumors are rare and constitute only 5% of all thoracic neoplasms [1]. Secondary chest wall tumors are much more common, with most arising from cancers of the lung and breast. Treatment of these neoplasms usually involves some degree of chest wall resection, which can be accompanied by complications such as visceral scarring, restrictive lung disease, and local deformity.

Scoliosis is a particularly well-known complication of chest wall resection [2]. Indeed, it has been reported that up to 99% of patients who undergo chest wall excision can suffer from some degree of scoliosis [3]. The literature on scoliosis after chest wall resection has provided many important insights into the natural history of the disorder. These include findings such as the convexity of the spine is always towards the side of resection, the rate of progression varies with the age of the patient at the time of surgery, and the number of resected ribs correlates with the degree of curvature that develops [3-7]. It has also been reported that resection of the anterior ribs rarely produces significant scoliosis, but resection of the posterior ribs frequently does [4,8]. 

In this case series, we review the results and the literature to discuss the likely etiology of the disorder while presenting seven clinical patients who underwent chest wall excision for the removal of a tumor and either developed scoliosis or underwent prophylactic spinal fusion and instrumentation to prevent scoliosis.

## Materials and methods

A retrospective chart review was performed of seven patients who underwent an extensive posterior chest wall excision for the removal of a chest wall tumor (Table 1). As these cases are exceedingly rare, the only inclusion criteria were patients with chest wall tumors who were surgical candidates in our clinic. In all cases, an experienced surgical oncologist performed the surgery. Five males and two females were included in the study, with a mean age of 35 years ± 25 (range 11-71 years). The types of chest wall tumors removed comprised of lung carcinoma in two patients, Ewing’s sarcoma, malignant fibrous histiocytoma, Wilm’s tumor, clear-cell sarcoma, and malignant schwannoma in one patient each.

**Table 1:** Posterior chest wall excision patients. F: female; M: male; T: post-reconstruction treatment; P: prophylactic treatment.

Patient #	Age (years)	Gender	Tumor	# Ribs resected	Post-resection Cobb angle	Treatment or prophylaxis	Levels fused	Post-fusion Cobb angle
1	71	F	Malignant fibrous histiocytoma	7 (ribs 3–9)	95	None	None	N/A
2	14	M	Wilm’s tumor	1 (rib 9)	94	T	T2-L4	46
3	11	M	Clear cell sarcoma	4 (ribs 5–8)	70	T	T2-L2	11
4	56	F	Malignant schwannoma	6 (ribs 7–12)	42	T	T3-L2	10
5	70	M	Lung cancer	3 (rib 7–9)	0	P	T5-T11	0
6	52	M	Lung cancer	3 (ribs 3–5)	0	P	T3-T9	0
7	32	M	Ewing’s sarcoma	3 (ribs 8–10)	5	P	T5-T12	5

## Results

Patient cases

**Figure 1:**
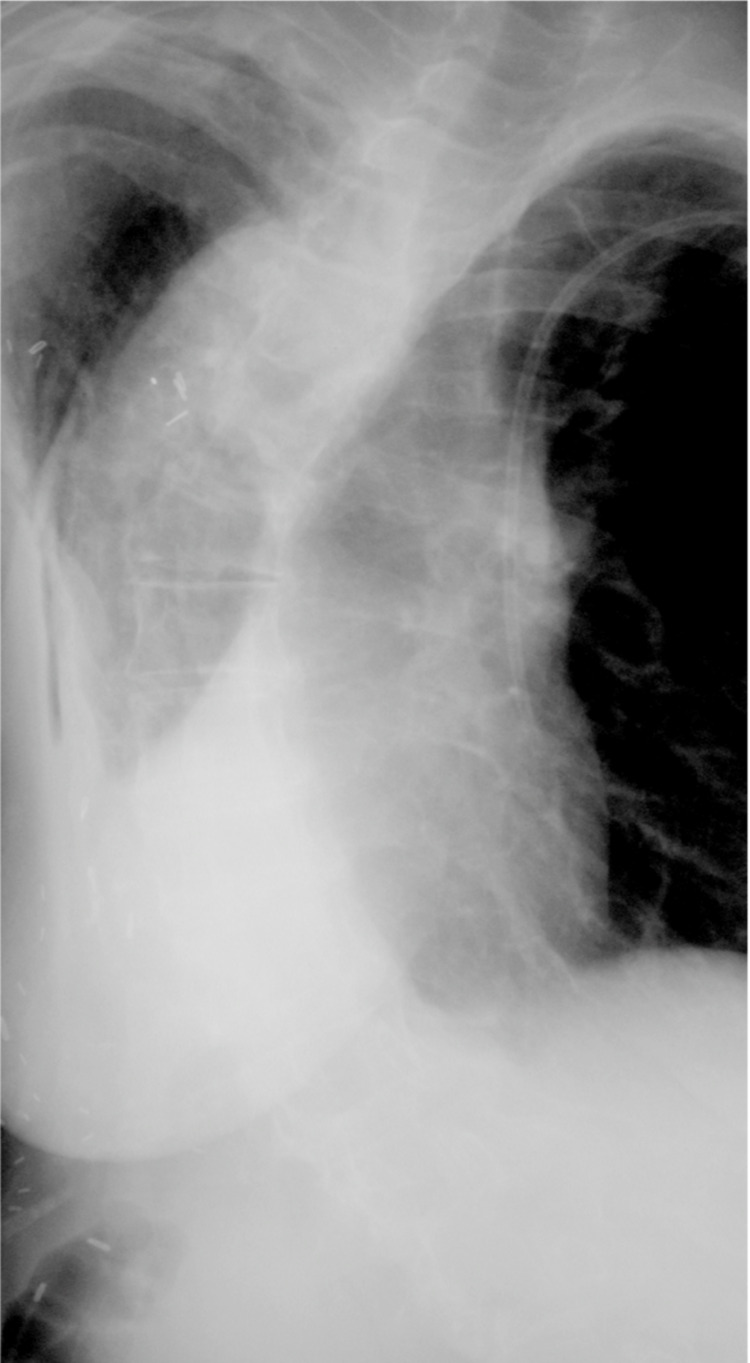
Posterior-anterior radiograph of the spine showing a left thoracolumbar curvature of 95 degrees from T2 to L3 following a chest wall resection of ribs 3 through 9 for a recurrent malignant fibrous histiocytoma.

Patient 1 was a 71-year-old woman who underwent a chest wall excision for the removal of a recurrent malignant fibrous histiocytoma that included disarticulation and partial posterior rib resection of left ribs 3 through 9. Subsequently, a progressive convex, left-sided thoracolumbar scoliosis developed, which measured 95 degrees from T2 to L3 (Figure 1). Options for management were discussed, but she elected non-operative treatment of her severe reduction in pulmonary function.

**Figure 2:**
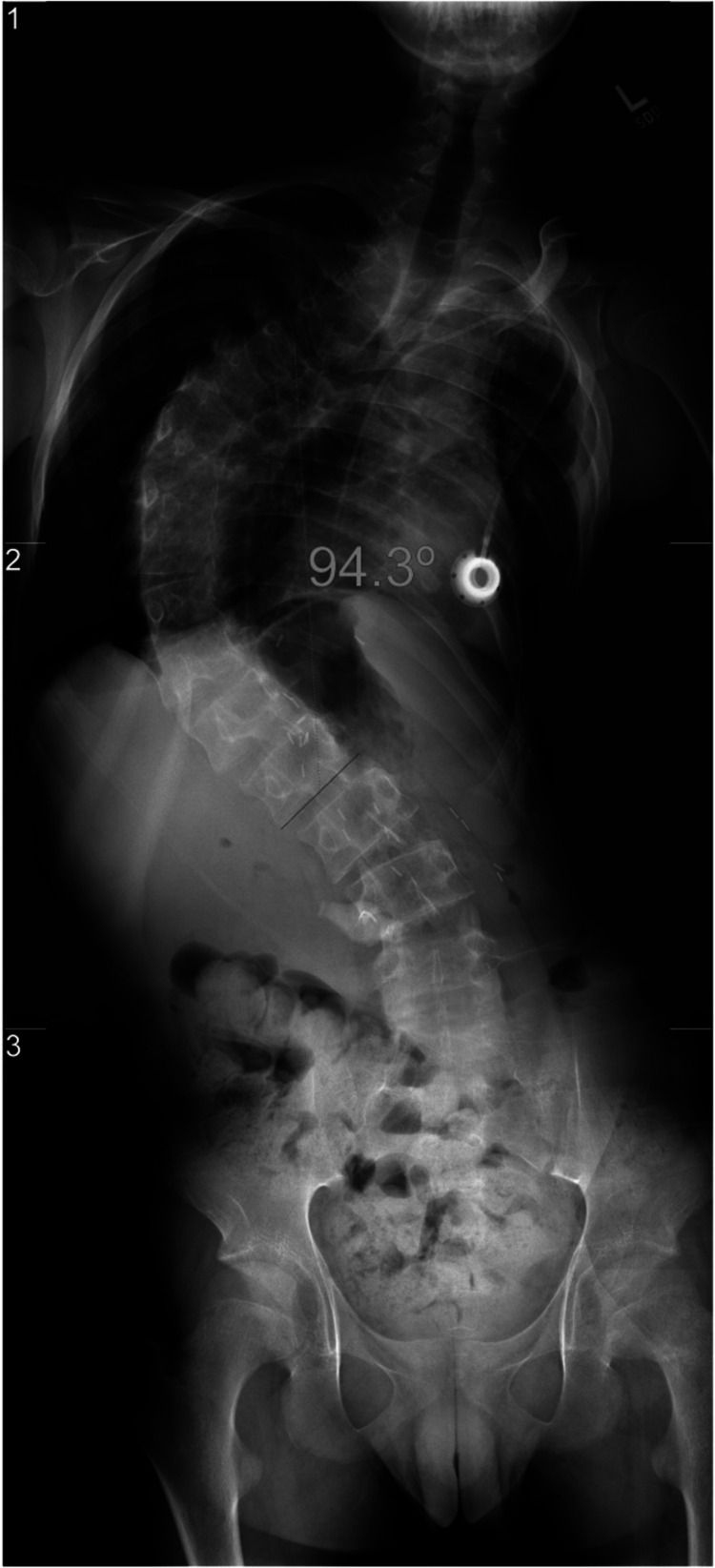
Preoperative posterior-anterior radiograph of the spine showing a right thoracolumbar curvature of 94 degrees from T4 to L2 following a chest wall resection of the ninth rib and radiation therapy.

**Figure 3:**
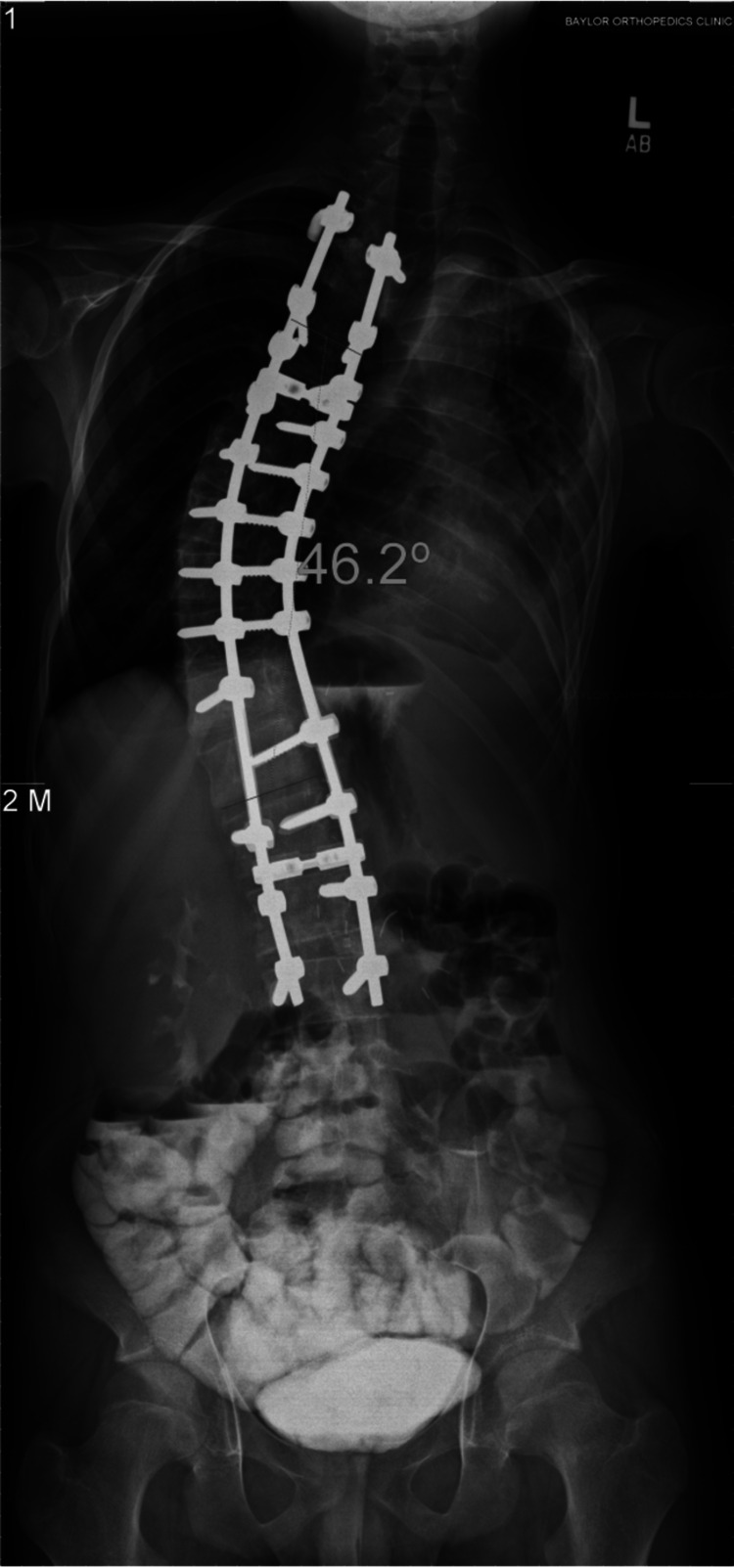
Postoperative posterior-anterior radiograph of the spine showing correction of the curve to 46 degrees in the patient shown in the previous figure.

Patient 2 was a 14-year-old male who presented with scoliosis following chest wall resection performed for the removal of a Wilm’s tumor. He had undergone neoadjuvant radiation therapy to shrink the tumor prior to the resection. The chest wall resection involved the removal of the posterior aspect of the right ninth rib. After surgery, he underwent adjuvant chemotherapy. Within two months of surgery, however, a convex right thoracolumbar scoliosis developed that measured 30 degrees. He was then placed in a thoracolumbar spine orthosis brace. However, the curvature continued to increase, and by 12 months after chest wall reconstruction, it had reached 94 degrees from T4 to L2 (Figure 2). He then underwent a T2 to L4 posterior spinal fusion and instrumentation with T4 to L4 Smith-Peterson osteotomies. Postoperatively, his curvature measured 46 degrees (Figure 3).

Patient 3 was an 11-year-old boy who presented with clear-cell sarcoma of the left lateral chest wall. He received neoadjuvant chemotherapy to shrink the tumor before a chest wall resection that included excision of the posterior aspects of ribs 5 through 8. Within six months of the resection, a convex left thoracolumbar scoliosis developed with a 25-degree Cobb angle. Scoliosis then progressed over the next 24 months, by which time it had reached 70 degrees from T1 to L1. He then underwent a T2 to L2 posterior spinal fusion and instrumentation, which corrected his curvature to 11 degrees from T1 to L1.

Patient 4 was a 56-year-old woman who had undergone excision of a right paraspinous malignant schwannoma from T10 to T12 at the age of 44. At the age of 52 years, the tumor recurred in the left paraspinous region. She then underwent excision of this second mass, which included resection of the posterior aspect of the left ribs 7 through 12. Radiographs obtained immediately after surgery did not show any evidence of scoliosis. However, a 23-degree left-sided scoliosis from T6 to T12 was noted within one month of resection. She was placed in a spinal orthosis brace, but the curvature continued to progress until it had reached 42 degrees within six months. Scoliosis was a long, sweeping C-shaped curve. Posterior spinal fusion with instrumentation from T3 to L2 was performed, which decreased the curve to 10 degrees from T3 to L2.

Patient 5 was a 70-year-old man with a locally invasive, right lower lobe, non-small-cell lung carcinoma that involved the lateral portion of the T8 vertebral body and ribs 7 to 9 on the right. He underwent right lower lobe removal, chest wall resection of ribs 7 through 9, and a transpedicular partial excision of the T7 through T9 vertebral bodies. Preoperatively, it had been determined that the patient would also undergo simultaneous spinal stabilization using anterior spinal instrumentation without fusion from T5 to T11 to prevent scoliosis from developing after chest wall excision. The patient did not develop scoliosis during follow-up; however, he died six months postoperatively of his lung carcinoma.

Patient 6 was a 52-year-old man who had a right upper lobe small-cell lung carcinoma that had invaded the posterior chest wall. A posterior chest wall excision with resection of ribs 3 to 5 and a right upper lobectomy were performed to remove the tumor. He underwent immediate prophylactic anterior spinal instrumentation from T4 to T7 and posterior spinal fusion and instrumentation from T3 to T9 to prevent spinal curvature. Postoperatively, scoliosis did not develop, but the patient died of the disease 11 months postoperatively.

Patient 7 was a 32-year-old man with a history of Ewing’s sarcoma of the vertebral bodies of T8 and T9 and of the ninth rib. He received neoadjuvant chemotherapy to shrink the tumor, which was followed by an en bloc excision of the chest wall. This involved resection of the posterior aspects of ribs 8 to 10, excision of the entire ninth rib, and en bloc resection of the T8 and T9 vertebral bodies. This was followed by prophylactic spinal stabilization with anterior spinal instrumentation from T7 to T10 combined with posterior instrumentation from T5 to T12. He showed 5 degrees of scoliosis postoperatively, but there has been no progression over the past seven years. 

Outcomes

From the seven case reports discussed, the number of ribs removed ranged from 1 to 7, with a mean of 3.33 ± 2.04. Three patients underwent prophylactic spinal fusion and instrumentation to prevent scoliosis after their chest wall reconstruction. Of these three patients, one had no scoliosis at seven years of follow-up and the other two died within a year of unrelated medical causes. Four patients who did not undergo prophylactic spinal fusion and instrumentation developed scoliosis after chest wall resection with a mean Cobb angle ranging from 42 to 95 degrees, with a mean of 71.58 ± 25 degrees. Three of these patients then underwent surgery to correct scoliosis, but the fourth patient declined further surgical intervention due to the high risk of cardiopulmonary complications associated with her comorbidities. The post-operative Cobb angles ranged from 10 to 46 with a mean of 17 ± 20.5 degrees.

## Discussion

The natural history of the scoliosis that commonly forms after chest wall resection has been well described by many authors [3-7]. Kawakami et al. reported that age is an important factor in determining the progressive nature of scoliosis. In their report, five of six children who underwent chest wall resection required spinal fusion to prevent the progression of scoliosis, whereas only one in five adults needed fusion to arrest the progression [4]. In contrast, we observed scoliosis developing in both our adult and pediatric patients who did not undergo prophylactic spinal fixation. The development of scoliosis after chest wall resection is likely multifactorial, including but not limited to location of resection, radiation therapy, laminectomy, and neuromuscular dysfunction [8-11].

On the other hand, Feiertag et al. performed an animal study in which the location of the rib resection was assessed in light of the resultant degree of curvature [12]. Two separate resection locations were compared: the rib head and neck versus the rib shaft, with mobilization of the ribs with no resection serving as the control. He showed that scoliosis developed to a greater degree following resection of the posterior (head and neck) rather than the anterior (shaft) aspect of the rib [3]. DeRosa similarly showed that posterior rib resection is more likely than anterior rib resection to cause severe scoliosis [5].

Other commonly observed characteristics reported include the fact that the convexity of the curve is always toward the side of the resection [3-5]. In addition, the more ribs resected, the greater the curvature [3,5-7]. Specifically, all patients in this case report underwent resection of the posterior aspect of the ribs, and scoliosis developed in all patients who did not undergo prophylactic instrumentation. In addition, scoliosis developed with the convexity toward the side of the resection in all patients who did not undergo prophylactic instrumentation. The patients who underwent prophylactic instrumentation did not develop scoliosis after surgery. 

The etiology of the scoliosis that forms after chest wall resection is unclear and likely multifactorial. Oda et al. performed cadaveric studies which showed that the costovertebral joints stabilize the thoracic spine in the sagittal, coronal, and transverse planes [13]. Watkins et al. found that the rib cage significantly increased the stability of the thoracic spine in flexion, extension, lateral bending, and axial rotation [14]. Therefore, it seems to follow that any disruption in the rib cage would compromise the stability of the spine. Furthermore, Pal hypothesized that the vertical stability of the thoracic spine is maintained by the equal support coming from the ribs on both sides and is due to the equal load delivered to the lamina by both ribs via the costotransverse articulations and ligaments [15]. Therefore, when one side is resected, the opposite side bears an increased load, leading to compression of the facet joints and intervertebral discs. Later, Pal et al. conducted biomechanical studies in rabbits that demonstrated the importance of the chest wall in maintaining the stability of the spine [16]. Deguchi et al. studied the effects of rib resection or transection on spinal alignment and found that scoliosis could be induced by the resection of ribs. However, transecting the ribs resulted in only healing at the transection site and no scoliosis [7]. These studies support the hypothesis that the scoliosis that develops after chest wall excision is in part mechanical in nature.

Resection of the structural support of the ribs probably results in asymmetric loading of the spine and subsequent deformity. Piggott performed posterior rib resection in 25 children with idiopathic scoliosis and found that resection on the concave side significantly retarded the progression of scoliosis [17]. Further support for this etiology comes from Deguchi et al., who showed that rib resection on the concave side of the curvature could correct or slow the progression of scoliosis [18]. 

Another possible cause of scoliosis after chest wall resection is neuromuscular in nature. Acquired spinal cord injury usually results in paralytic scoliosis. The pathogenesis involves denervation of the musculature of the body. Without working musculature, the spine is then unable to maintain proper alignment. During resection of the chest wall, the neurovascular structures that traverse along the undersurface of the ribs are typically ligated, which results in denervation of the paraspinal muscles and the musculature of the chest wall. The paralysis of these muscles and the loss of the structural support of the ribs probably contribute to the development of scoliosis after chest wall excision. The result is a convex curvature toward the ipsilateral side of the resection.

There are few reports of the treatment of scoliosis after chest wall excision. Posterior spinal fusion with instrumentation has been the standard of care for the treatment of scoliosis since the early 1960s [19]. Since that time, many proponents of anterior fusion and instrumentation have shown satisfactory results from this strategy [20-23]. However, most surgeons agree that anterior surgery is technically more difficult in patients with more severe curves or in patients who have undergone previous thoracic surgery. Therefore, prevention of the scoliosis through the prophylactic placement of anterior spinal instrumentation at the time of chest wall excision may be the best course of action. The placement of anterior spinal instrumentation with or without fusion is facilitated at this time by the extensive exposure of the vertebral bodies and discs once the chest wall is excised. In addition, placement of anterior instrumentation is relatively straightforward at this time because normal spinal anatomy is present and there is no deformity or adhesions of the visceral or vascular structures to the spine. Moreover, posterior instrumentation via a simultaneous anterior and posterior approach in the lateral decubitus position can be performed if necessary. Combined posterior and anterior instrumentation has been shown to give the greatest immediate rigidity in flexion, extension, and rotation [24].

Importantly, progressive scoliosis developed in none of the three patients in our current series who underwent prophylactic spinal fusion and instrumentation. Performing a prophylactic fusion not only prevents the development of scoliosis but can also save lumbar levels of fusion compared to patients operated on later with a large Cobb angle. None of our cases where prophylactic fixation was performed required fusion to the lumbar spine, whereas cases that required corrective surgery involved extension into the lumbar spine and multiple posterior column osteotomies. The average number of fused segments for prophylactic fixation was 6.33 ± 0.58, with an average post-operative Cobb angle of 1.6 degrees ± 2.89. In comparison, the average number of fused segments for corrective surgery was 12.27 ± 1.53, with an average post-operative Cobb angle of 17.17 degrees ± 20.5. 

## Conclusions

To the best of our knowledge, there are currently no studies that have examined prophylactic spinal stabilization in patients who undergo chest wall resection. The natural history of scoliosis after chest wall resection is not immediate, but rather one of progression. The etiology of the scoliosis that develops after chest wall excision is probably both mechanical and paralytic in nature. Our results suggest that the prophylactic treatment of scoliosis after chest wall resection decreases the risk of scoliosis. Prophylactic surgery has the benefit of saving motion segments as well as decreasing the overall surgical burden on the patient and the surgeon. In the words of Benjamin Franklin, an ounce of prevention is worth a pound of cure.
